# Epigenetic Alterations in Alzheimer’s Disease: Impact on Insulin Signaling and Advanced Drug Delivery Systems

**DOI:** 10.3390/biology13030157

**Published:** 2024-02-28

**Authors:** Alosh Greeny, Ayushi Nair, Prashant Sadanandan, Sairaj Satarker, Ademola C. Famurewa, Madhavan Nampoothiri

**Affiliations:** 1 Department of Pharmacology, Manipal College of Pharmaceutical Sciences, Manipal Academy of Higher Education, Manipal 576104, India; alosh.mcopsmpl2022@learner.manipal.edu (A.G.); sairaj.satarker@learner.manipal.edu (S.S.); 2 Department of Pharmaceutics, Amrita School of Pharmacy, Amrita Vishwa Vidyapeetham, Amrita Health Science Campus, Kochi 682041, India; ayushinair08@gmail.com; 3 Department of Pharmaceutical Chemistry, Amrita School of Pharmacy, Amrita Vishwa Vidyapeetham, Amrita Health Science Campus, Kochi 682041, India; prashants@pharmacy.aims.amrita.edu; 4 Department of Medical Biochemistry, Faculty of Basic Medical Sciences, College of Medical Sciences, Alex Ekwueme Federal University, Ndufu-Alike, Ikwo 482123, Nigeria; ademola.famurewa@funai.edu.ng

**Keywords:** pigenetics, Alzheimer’s disease, DNA methylation, histone deacetylase, insulin signaling, drug delivery

## Abstract

**Simple Summary:**

This review mainly focuses on epigenetic changes in a few selective genes associated with insulin insensitivity in the periphery and how this peripheral insulin insensitivity is linked with impaired signaling of insulin in the brain, thus causing Alzheimer’s disease. DNA methylation and histone modifications are the focus of this review, with primary importance given to DNA methylation. Moreover, there has been a focus shift from the amyloid β hypothesis to epigenetic mechanisms. Furthermore, we discuss the advanced drug delivery systems that can be used for the delivery of drugs targeting the brain during Alzheimer’s disease. The advanced drug delivery systems discussed in this paper are nanoparticles, vesicular systems, network systems and dendrimers, hydrogel-based systems, and biologics.

**Abstract:**

Alzheimer’s disease (AD) is a neurodegenerative condition that predominantly affects the hippocampus and the entorhinal complex, leading to memory lapse and cognitive impairment. This can have a negative impact on an individual’s behavior, speech, and ability to navigate their surroundings. AD is one of the principal causes of dementia. One of the most accepted theories in AD, the amyloid β (Aβ) hypothesis, assumes that the buildup of the peptide Aβ is the root cause of AD. Impaired insulin signaling in the periphery and central nervous system has been considered to have an effect on the pathophysiology of AD. Further, researchers have shifted their focus to epigenetic mechanisms that are responsible for dysregulating major biochemical pathways and intracellular signaling processes responsible for directly or indirectly causing AD. The prime epigenetic mechanisms encompass DNA methylation, histone modifications, and non-coding RNA, and are majorly responsible for impairing insulin signaling both centrally and peripherally, thus leading to AD. In this review, we provide insights into the major epigenetic mechanisms involved in causing AD, such as DNA methylation and histone deacetylation. We decipher how the mechanisms alter peripheral insulin signaling and brain insulin signaling, leading to AD pathophysiology. In addition, this review also discusses the need for newer drug delivery systems for the targeted delivery of epigenetic drugs and explores targeted drug delivery systems such as nanoparticles, vesicular systems, networks, and other nano formulations in AD. Further, this review also sheds light on the future approaches used for epigenetic drug delivery.

## 1. Introduction

AD is a progressively developing neurodegenerative condition mainly targeting the hippocampus, which involves neural circuits of memory processing and consolidation. AD pathology is characterized by intracellular neurofibrillary tangles (NT) and neuritic plaques due to excessive buildup of extraneuronal Aβ peptide in the neocortical regions and the medial temporal lobe. About one tenth of the individuals in the geriatric population beyond the age of sixty-five and four tenths of the individuals who are above eighty-five years of age are diagnosed with AD. By 2050, it is expected that the confirmed cases of AD will drastically increase [1]. AD is named after Alois Alzheimer, a German psychiatrist, who noticed the presence of an excess amount of amyloid plaques in the brain of a patient who previously suffered memory loss before death [2]. AD neuropathology involves positive and negative lesions. Positive lesions are due to the aggregation of deposits such as NT, dystrophic neurites, amyloid plaques, and other deposits in certain parts of the brains of AD patients. In contrast, negative lesions include the loss of neural, synaptic, and neuropil, leading to large atrophy [3,4,5]. A few tests for diagnosing AD for suspected patients include magnetic resonance imaging (MRI) for neurons, neurological examination, vitamin B_12_ deficiency, and patient examination [6,7].

AD can be categorized into early onset AD (EOAD), which is associated with cognitive impairment and develops before reaching the age of sixty-five years, and late onset AD (LOAD), associated with cognitive impairment and develops after sixty-five years of age. This stage accounts for more than 90% of confirmed AD patients [8]. The four clinical stages in AD are as follows: preclinical/presymptomatic, characterized by mild memory loss and absence of clinical signs [9,10,11]; early-stage AD, where concentration and memory loss become more frequent with slow depression [11,12]; moderate AD stage associated with severe memory loss and late-stage AD called the severe form of AD [11].

Several studies and research were conducted in view of the Aβ hypothesis to explore the etiology of AD and to find a treatment for AD. However, the conducted studies were unable to explain fully the pathogenesis of AD [13]. Waddington, in 1942, used the term “epigenetics” to illustrate the genetic control of embryonic development [14,15]. Some studies have put forward the argument that AD may not just be an advanced stage of aging but instead a disruption in the natural process of aging. Moreover, the natural process of aging may also protect against AD where epigenetics is involved [16]. Looking into the cellular level changes in an AD brain, various molecular pathways and intracellular signaling are dysregulated, which include Aβ and tau, inflammatory-immune responses, homeostasis and plasticity of synapses, and oxidative stress and their dysregulation results from genetic, environmental, and biologic interventions [17,18]. Furthermore, accumulating evidence has been pointing towards the possibility of an imbalance in epigenetic mechanisms responsible for abnormal synaptic plasticity and memory-associated genes in AD [19,20,21,22,23].

The role of insulin in maintaining glucose levels in liver, muscles, and the adipocytes, is well recognized. Recent research demonstrates that AD has been linked to aberrant brain insulin receptor signaling [24,25]. This implies the importance of comprehending how insulin/insulin receptor signaling affects neurodegeneration.

In this paper, we focus more on how epigenetic mechanisms alter the peripheral and central insulin signaling, leading to AD as well as a few important drug delivery systems which may be useful in combating the epigenetic changes responsible for AD.

## 2. Epigenetics

Epigenetics is the mitotically or meiotically heritable traits observed in gene functions due to alterations in the chromosome, which cannot be elucidated by modifications in DNA sequence [26,27]. Although there is no direct alteration in DNA patterns, the gene expression levels are altered due to a few epigenetic changes that include chemical modifications to the DNA bases and changes to the chromosomal structure where the packaging of DNA takes place [28]. One of the critical features of epigenetics is that they are inherited between mother and daughter cells, commonly referred to as mitotic inheritance, and between generations, commonly known as meiotic inheritance. Epigenetics explains how some organisms with identical DNA can have vast phenotype differences [29].

### 2.1. DNA Methylation

This is one of the most common mechanisms of epigenetic modification where a methyl group is added covalently from the S-adenyl methionine (SAM) to the cytosine-C5 position, the entire process being driven by DNA methyl transferases (Dnmts) to form 5-methylcytosine (5mC). Dnmt3b and Dnmt3a are also referred to as de novo Dnmts, which could establish new methylation patterns in unmodified DNA. At the same time, during DNA replication, Dnmt1 is involved in transferring the methylated sequence from the parent strand to the freshly produced daughter strand. As the cells approach terminal differentiation, Dnmt expression is reduced. Even though the highest levels of methylation in DNA occur in the brain, it makes up for hardly 1% of nucleic acids in the human genome [30,31]. DNA methylation was earlier thought to play the silencing role, where increased methylation levels were correlated with reduced expression [32]. Methylation of cytosines can occur at sites other than CpG and is involved in regulating the gene expression in embryonic stem cells [33]. Further, micronutrients like zinc have a significant impact on enzymes such as methionine synthase, and its deficiency may disrupt DNA methylation and histone modification [34].

### 2.2. Histone Modifications

Histones are those proteins to which the DNA strands wind up. These are further composed into structures called nucleosomes. The four types of histones that are important for the structural organization of DNA are H2A, H2B, H3, and H4 [35]. Histone modifications include a wide variety of modifications that happen after the translation process and regulate the chromatin structure. These include methylation, ubiquitination, phosphorylation, and acetylation [36]. The ‘histone code hypothesis’ states that DNA transcription is primarily controlled by a combination of post-translational modifications to the histone proteins [37].

Histones organize themselves as a unit of eight molecules called octamers. The DNA, which is negatively charged, winds around the positively charged histone octamer to form a nucleosome. The nucleosome contains one hundred and forty-seven base pairs of DNA associated with an octameric histone protein core. The two sets of chromatin are euchromatin and heterochromatin. Euchromatins are responsive to transcription, whereas heterochromatins are compact structures that are not transcribed [35,38].

Histones are protein structures consisting of an N-terminus and a C-terminus, comprising approximately thirty percent of the total mass of the histone protein. The histone tails protrude out from the chromatin surface, providing a higher surface area for chemical modifications to take place. These tails consist of the amino acid lysine, which supports the chemical modifications of methylation and acetylation. Histone methylation occurs when a -CH_3_ group is covalently added to the lysine residue and has a repressive action, leading to a heterochromatin state. In contrast, histone acetylation occurs when an acetyl group is added to the ε-amino lysine residue in H3 and H4 tails, which results in a euchromatin state [35,39]. Histone acetyltransferases (HAT) and its co-factors, such as CREB binding protein (CBP), GNAT, MYST, and p300, are recruited by DNA sequence-specific transcription factors that identify and target those regions of chromatin where acetylation takes place [40].

### 2.3. Non-Coding RNA

Non-coding RNA (ncRNA) is an epigenetic mechanism whereby a biologically active RNA molecule has undergone transcription but does not get translated or encode a protein [41]. ncRNAs include dicer-dependent microRNAs (miRNA) and short-interfering RNAs (siRNA), shaped by RNA interference pathways [42]. Literature suggests the presence of microRNAs in astrocytes that regulate glutamatergic and inflammatory signaling, which affect synaptic plasticity [43]. siRNAs include nucleotides less than thirty in number, while long non-coding RNAs (lncRNA) include more than two hundred nucleotides [44]. Apart from their role in gene silencing, reports suggest their role in DNA methylation and histone modifications.

## 3. Insulin and Epigenetic Mechanisms

Insulin is an anabolic hormone released by the pancreatic β-cells present which gets activated with the rise in glucose levels in the blood. The blood glucose level is reduced by insulin by boosting the uptake of glucose and favoring glycolysis in skeletal and adipose tissues. GLUT4 is a glucose transporter that does not require ATP and mediates glucose uptake from the blood. This kind of transfer is classified as facilitated diffusion [45,46]. Insulin also enhances the process of glycogen synthesis in skeletal muscle, adipose tissue, and liver [47]. Within the insulin promoter Cis-acting regulatory elements have demonstrated the ability to bind to a wide variety of transcription factors. Among these, cyclic adenosine monophosphate (cAMP) responsive element (CRE) is one of the regulatory elements that have the capability to bind to a wide array of transcription factors [48]. There are four CREs in humans, while in the case of rodents, only one site of CRE exists. Among these, CRE2 remains the only CRE conserved within humans and rodent species. cAMP responsive binding protein-1 (CREB-1) is a CRE-associated transcription factor having an inhibitory effect on transcription while activating transcription factor-2 (ATF-2), as the name suggests, activating transcription. Studies report that CRE2 mutation inhibits the ATF-2 effect and plays a significant part in gene regulation [48,49].

Furthermore, studies have shown that there are nine CpG sites at regions upstream to the transcription start site (TSS) in human insulin (INS) promoters, whereas there are three CpG sites upstream to the transcription site of mouse Ins2 promoters. Detailed studies showed unmethylated CpG sites in β-cells of human INS and mouse Ins2, in contrast to the other tissues. These methylated CpG sites were shown to be responsible for suppressing ninety percent expression of insulin promoter-driven reporter genes [50].

More studies were conducted to evaluate how methylation events impact the overall expression of the insulin gene. The CpG sites that appeared in the mouse Ins2 promoters were methylated individually, as a result of which insulin promoter activity was suppressed by fifty percent. These results prove that along with methylation, there may be other mechanisms contributing to suppressing the insulin gene expression. Upon further analysis, it was observed that the methylation of CRE CpG hindered the in vivo interaction of CREB and ATF-2 with Ins2 in NIT-1 cells and increased the interaction of methyl CpG binding protein 2, thus decreasing the expression of insulin gene [50]. In vitro studies in Ins2 embryonic stem cells showed that initial methylation occurs in the insulin gene; nevertheless, upon maturation, it gets demethylated, resulting in differentiation into β-cells, producing insulin, thus suggesting the significant contribution of demethylation in the maturation of β-cells and insulin gene expression in specific tissues [50]. Suppressing the gene transcription at the INS gene promoter, specifically at CpG islands via DNA methylation, is an essential element that shapes the developmental process of type 1 diabetes [51]. These islands are frequently observed near the TSS. Dnmts drive the cytosine methylation. Dnmts include DNMT1, DNMT3A, and DNMT3B. These members are expressed in large numbers during the progression of diabetes due to DNA methylation [52]. Methylated DNA enters the circulation following the β-cell destruction by cytotoxic T-lymphocytes [53]. Moreover, another study, that examined the role of DNA methylation using pancreatic islet cells from type 2 diabetes mellitus (T2DM) and non-T2DM donors, reports a reduction in insulin secretion stimulated by glucose, expression of insulin mRNA, and assessing insulin concentrations in the pancreatic islets of both the donor groups. A rise in the levels of DNA methylation at four CpG sites, namely −234, −180, −102, and +63, was observed in T2DM pancreatic islets. At sites −234, −180, and +63, the degree of methylation and mRNA expression of insulin were negatively correlated. Additionally, the consequence of hyperglycemia on methylation levels at the promoter region was also investigated. Two CpG sites, namely −1057 and +58 in the INS gene promoter, were reported to be hypermethylated [54]. In a study involving Zucker diabetic fatty rats, the outcome of excessive nutrition on DNA methylation was analyzed. It was found that the expression of Dnmt1 mRNA had a direct correlation with higher glucose concentrations. As a result, five CpG positions present within the promoter region of Ins1 had higher methylation levels. These included CRE and the downregulation of Ins1 mRNA synthesis [55]. A rise in the methylation levels at the promoter region of Ins1 was observed in the islets in the pancreas isolated from Zucker diabetic fatty rats. Moreover, artificial methylation of the *Ins1* promoter was reported to suppress the luciferase activity. Furthermore, it was observed that DNA methylation inhibitors reversed the methylation of the *Ins1* promoter and showed a significant decline in the methylation levels [55].

## 4. DNA Methylation and Insulin Resistance

DNA methylation is the process of introducing a methyl (-CH_3_) group to the 5′-position of the cytosine within 5′-CpG-3′ covalently. This addition creates a 5′-methyl cytosine [56]. CpG sites arrange themselves in such a way to make up repetitive sequences known as CpG islands [57]. For transcription to take place in a gene, the accessibility of the promoter and the regulatory sites play a very crucial role. This introduction of the -CH_3_ groups decreases the accessibility of the DNA and prevents the DNA from becoming bound by the transcription factors, thus altering the gene expression [58]. Therefore, CpG island hypermethylation results in the silencing of transcription, whereas hypomethylation leads to the initiation of transcription [59].

### 4.1. DNA Methylation at IRS1/2

Insulin receptors become activated upon insulin binding to its receptor, followed by which, the insulin receptor substrates IRS1/IRS2 are brought to the plasma membrane [60]. In the liver, IRS2, which is present in the hepatocytes, regulates insulin signaling.

In a study conducted on mice, it was reported that the destruction of IRS2 does not cause IR in skeletal muscle, but the results were the opposite in the case of the liver [61]. A study done by Krause et al. on individuals with T2DM reported a reduction in the extent to which IRS2 is expressed in the liver of individuals with obesity and T2DM, and this downregulation of IRS2 correlated with DNA methylation of hepatic IRS2 at the CpG5 island present near the promoter region [62]. The same study also reported a hypomethylated CpG-containing binding interface for sterol regulatory element binding transcription factor 1 (SRBF1), which was previously reported to contribute to the repression of IRS2 promoter [63].

### 4.2. DNA Methylation at PPARGC1A

Peroxisome proliferator-activated receptor gamma coactivator 1α (PGC1α) is a transcriptional coactivator that is induced by fasting and is encoded by the PPARGC1A gene. It is responsible for maintaining mitochondrial biogenesis [64]. The disruption of PGC1α has contributed to hepatic IR [65], and it was further observed that its affinity is higher at locations of MASLD-associated modifications in DNA methylation [66]. In a study carried out on fatty liver, the plasma insulin levels at fasting and the HOMA-IR correlated with the ratio of methylated to unmethylated DNA for liver PPARGC1A, whereas the ratio of methylated to unmethylated DNA for mitochondrial factor transcription factor A had an inverse correlation with IR [67].

### 4.3. FADS2

FADS2 gene encodes fatty acid desaturase 2(FADS2) [68] and metabolizes fatty acids through epigenetic modifications [69]. Accumulating genome-wide association studies have linked FADS2 and IR-related diseases [70,71,72]. SREBP1c is a major transcription factor regulating lipogenesis and lipid homeostasis [73]. FADS2-deficient animals showed the presence of disrupted SREBP1c and were also involved in the over-production of alternate enzymes accountable for the metabolism of fatty acids and, thus, IR [73]. Furthermore, there exists an inverse correlation between the expression of FADS2 and the activity of FADS2 in the serum, with the two CpG positions present in a neighboring enhancer becoming methylated along with the CpG-rich region present upstream to the FADS2 TSS. This indicates that the degree of methylation significantly contributes to modulating the FADS2 activity [74].

### 4.4. DNA Methylation and Insulin-Like Growth Factor Binding Proteins

**Figure 1:**
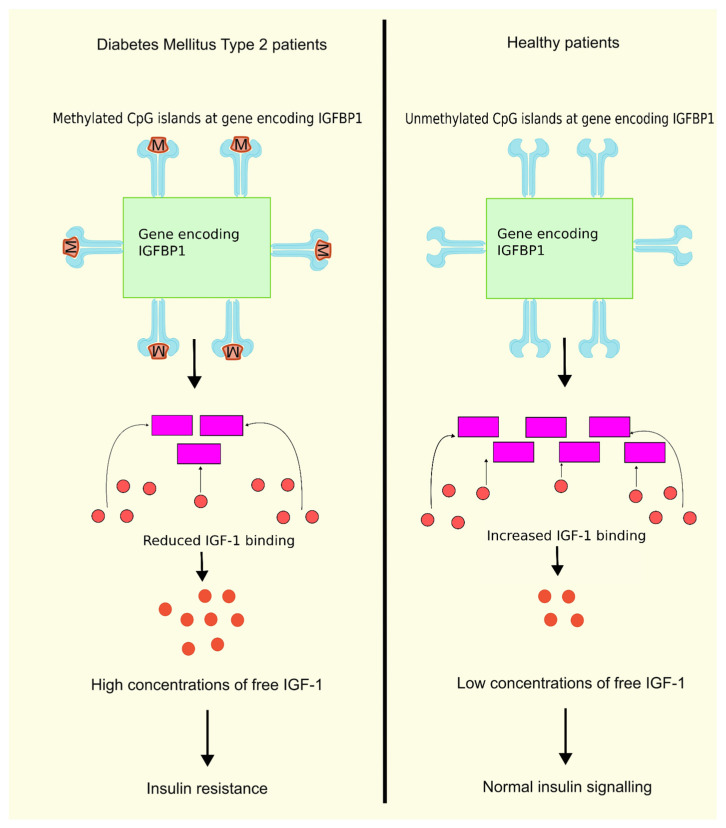
DNA methylation of IGFBP1 and insulin resistance. DNA methylation of the gene encoding IGFBP1 leads to lower serum levels of IGFBP1, reducing the binding of IGF-1 to IGFBP1, further leading to higher concentrations of free IGF1, and finally resulting in IR compared to normal unmethylated DNA having normal insulin signaling due to binding of IFG-1 to IGFBP1. When IGF-1 binds to IGFBP1, the concentration of free IGF-1 decreases, resulting in normal insulin signaling. Created using inkscape.org (Accessed on 24 February 2024).

Insulin-like growth factor binding protein 1 (IGFBP1) is one among the family of structurally homologous proteins that attaches to IGF-1 with good affinity and serves as a transport protein for IGF-1. The family consists of six IGFBPs, namely, IGFBP1, IGFBP2, IGFBP3, IGFBP4, IGFBP5, and IGFBP6. There are, however, other IGFBPs that are misnomered, such as IGFBP7, IGFBP8, IGFBP9, etc. Due to their high binding affinity to IGFs and their conserved protein structure, the IGFBPs 1–6 are considered true IGFBPs [75]. An inverse correlation exists between the IGFBP1 levels and the free IGF1 levels. A reduction in the circulating glucose and free IGF1 levels is achieved by administering exogenous IGFBP1. Previous research showed a decline in the level of IGFBP1 in T2DM patients, whereas the free IGF1 levels were increased in T2DM patients. It was also observed that IR is linked with reduced production and circulating levels of IGFBP1 [76]. In another study on T2DM patients, higher levels of DNA methylation were observed in six CpG positions in the gene encoding IGFBP1 compared to the control group. Moreover, IGFBP1 gene methylation levels were greater in patients diagnosed with T2DM having a familial history of the disease compared to patients without a familial history of this disease [77], as shown in Figure 1. These findings are conclusive that higher levels of DNA methylation in the IGFBP1 gene can lead to lower serum IGFBP1 levels, leading to higher concentrations of free IGF-1, thus resulting in IR and T2DM.

### 4.5. DNA Methylation and IGFBP 7

Insulin-like growth factor binding protein 7 (IGFBP7), encoded by the IGFBP7 gene, belongs to the IGFBP superfamily. It was one of the first IGFBP-related proteins discovered and is also designated as IGFBP-rp1 [78]. Studies on newly diagnosed men with T2DM exhibited increased levels of methylation at three CpG positions on IGFBP7 in comparison with the control group. However, the serum contents of IGFBP7 were similar across all the groups, i.e., the control group, the treated T2DM group, and the newly diagnosed T2DM group. They did not have any correlation with IGFBP7 DNA methylation levels. Moreover, the levels of IGFBP7 in the serum had a positive correlation with serum IGFBP1 levels, which is a marker to produce insulin found in men but not in women. In conclusion, low IGFBP7 levels may be linked with T2DM IR [79].

### 4.6. The Role of Dnmt3a in Insulin Resistance

DNA methyltransferases are a family of five members, namely Dnmt1, Dnmt2, Dnmt3a, Dnmt3b, and Dnmt3l [80]. Dnmt1 maintains DNA methyltransferase and mainly methylates hemimethylated DNA. Dnmt3a and 3b are called de novo DNA methyltransferases since they preferably act on unmethylated DNA. Dnmt3l lacks catalytic activity but is homologous to other Dnmt3s. Dnmt2 does not methylate DNA but methylates cytoplasmic tRNA [80]. In genetic knock-out studies, it was shown that administration of a Dnmt inhibitor demethylates the *Adipoq* promoter, improving insulin sensitivity [81].

**Figure 2:**
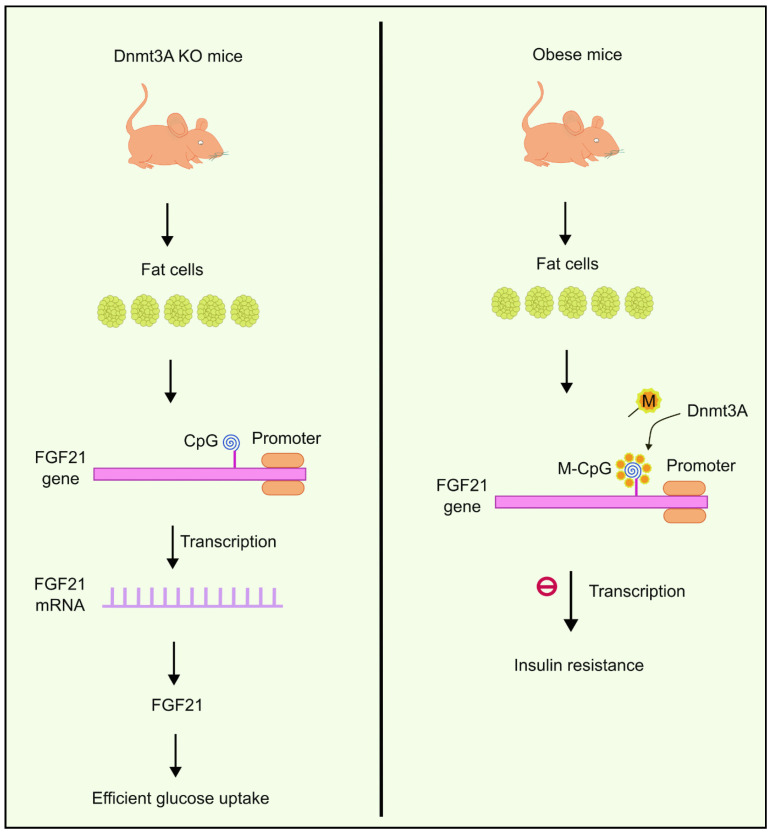
Overexpression of Dnmt3a inhibits the transcription of the FGF21 gene, which is responsible for glucose uptake to fat cells. Dnmt3a KO mice, on the other hand, were observed to have efficient glucose uptake. Created using inkscape.org (Accessed on 17 November 2023).

You et al. observed the existence of abnormally elevated levels of Dnmts in fat cells extracted from obese mice. One of the DNA methyltransferases, Dnmt3a, has been shown to contribute to developing IR. Dnmt3a knock-out mice had a better insulin-dependent glucose uptake profile compared to the wild type, and the results were like those of genetically engineered mice lacking Dnmt3a in their fat cells. It was also observed that overexpression of Dnmt3a had an inhibitory effect on the transcription of another gene called Fgf21 [82]. Fgf21 encodes secreted proteins that are essential in absorbing glucose to fat cells [83]. Based on this evidence, You et al. suggested the role of Dnmt3a in causing IR in the Fgf21 promoter via the methylation of CpG islands. Reduced expression of Fgf21 causes IR in fat cells. These results were also consistent with studies performed on human beings [82]. Higher methylation levels were observed near the Fgf21 gene (Figure 2) in diabetic individuals compared to people without diabetes. Moreover, the degree of methylation had an inverse correlation with expression levels of Fgf21 mRNA [82].

### 4.7. DNA Methylation and Glucokinase

**Figure 3:**
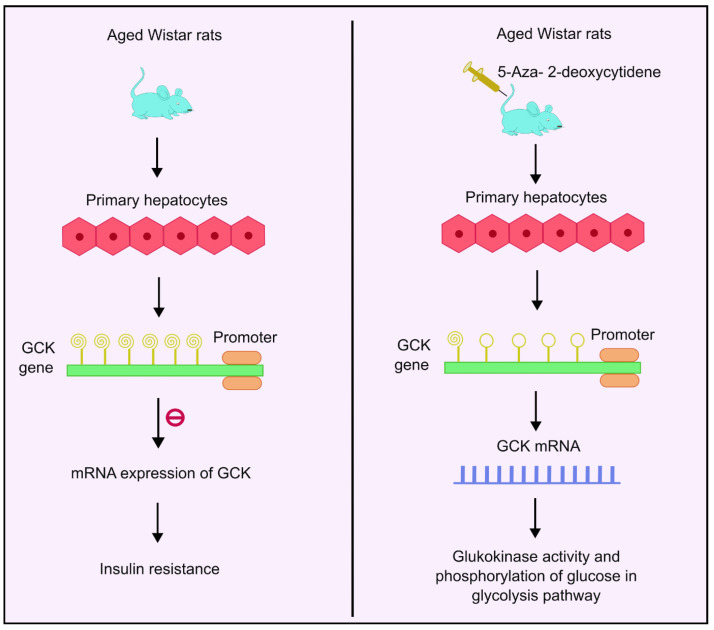
DNA methylation of GCK gene and insulin resistance. Higher methylation levels of the GCK gene inhibit mRNA expression of GCK, which is responsible for glucokinase activity and phosphorylation of glucose, thus leading to insulin resistance. Administration with 5-aza-2-deoxycytidene enhances the GCK expression by four times, thus leading to enhanced mRNA expression followed by higher glucokinase activity and phosphorylation of glucose in the glycolysis pathway. Created using inkscape.org (Accessed on 17 November 2023).

The glucokinase (GCK) gene regulates glucose homeostasis in humans by catalyzing phosphorylation of glucose. It acts as the rate-determining step in the glycolysis pathway in liver and pancreas [84]. It is a glucose sensor that helps maintain normoglycemia [85]. GCK is mainly concentrated in the liver and the pancreas [86]. Insulin stimulates GCK expression, which further activates glycolytic genes to increase glucose utilization. IR has been reported in T2DM patients with downregulated GCK activities [87]. Studies conducted in aged Wistar rats showed age-related methylation changes. As the aging progresses, the degree of methylation also increases, and a decline in GCK mRNA expression and glucokinase activity was observed. Analysis of 11 CpG sites showed increased methylation levels as the age progresses. Additionally, 5-aza-2-deoxycytidine, a DNA methyl transferase inhibitor, was used as the treatment. Administration of this drug enhanced the GCK expression four-fold in rat primary hepatocytes, as shown in Figure 3. Moreover, the analysis also showed that the age-related increased methylation of GCK in the liver had an inverse correlation with its expression, implying that there may be links between methylation and age-dependent hepatic IR [87].

### 4.8. DNA Methylation and Glucagon like Peptide 1

Glucagon-like peptide-1(GLP-1) is a thirty to thirty-one amino acid-long peptide hormone produced by the α-cells located in the pancreas. Along with the pancreas, enteroendocrine L cells, which are present in the intestines as well as the central nervous system, also secrete GLP-1 [88]. GLP-1 is mainly responsible for maintaining glucose homeostasis [89]. Studies conducted on rats and humans with T2DM have reported a decline in the presence of GLP-1 receptors. Studies have been conducted to link DNA methylation and GLP-1 mRNA expression. Pancreatic islets were isolated from adults who were grouped into T2DM and non-T2DM, and the expression levels for mRNA of Dnmts-1, 3a, and 3b, along with MECP2, were analyzed. In addition, a few CpG methylation levels were associated with the GLP-1 TSS. Further analysis of CpG positions at the +199 and +205 base pairs showed a reduction in glucose homeostasis and the presence of GLP-1 receptors in the islets isolated from T2DM donors. A slight increment in the methylation levels in T2DM islets was also reported but was not significant. Additionally, the methylation levels analyzed at the GLP-1 receptor promoter receptor were analyzed using α- and β-cells isolated from pancreatic cells. The DNA methylation levels at position 376 of the CpG site at the GLP-1 receptor were significantly elevated in α-cells compared to β-cells, and this had an inverse correlation with GLP-1 receptor expression [90].

### 4.9. Histone Modifications: Acetylation and Deacetylation

Histone proteins undergo acetylation and deacetylation via the action of HAT and histone deacetylases (HDACs), respectively. These modifications contribute a vital role in the epigenetic modulation of transcription [91]. When acetylation occurs in histones, it mainly relaxes the DNA and results in the initiation of transcription, while deacetylation, on the contrary, condenses the DNA protein and represses transcription [92]. In humans, there exist four main classes of histone deacetylases. These include class Ⅰ HDACs, which comprises HDACs 1–3 and 8; class Ⅱ HDACs can be further categorized into two subclasses—class Ⅱa and class Ⅱb HDACs. Class Ⅱa comprises HDACs 4, 5, 7, and 9, while class Ⅱb comprises of HDACs 6 and 10. Class Ⅲ HDACs are alternatively recognized by the term sirtuins. They include seven classes of sirtuins from SIRT1 to SIRT7. On the other hand, HDAC11 represents class Ⅳ HDAC [93].

HDACs, regardless of their class, take part in a vital role in regulating insulin signaling. Previous research showed that there have been interactions between HDAC2 and IRS-1 in yeast and ob/ob mice [94]. This interaction further leads to diminished acetylation and tyrosine phosphorylation (insulin-mediated) of the IRS-1 protein [94]. Trichostatin A is an antifungal antibiotic that selectively and reversibly inhibits mammalian class Ⅰ and Ⅱ HDACs [95]. This causes acetylation of IRS-1 and attenuation of IR [94]. Similarly, the role of HDAC3 in IR was also studied. A clinical study on 568 subjects in China demonstrated a significant association between HDAC3 and T2DM [96]. The study results were consistent with previous research done by Sun and colleagues that showed deletion of HDAC3 in the liver of C57BL/6 mice, resulting in hepatosteatosis and enhanced insulin responsiveness [97]. HDAC3 is also known to take part in modulating PPARγ function in adipocytes [98]. These studies have shown the role of HDAC3 in regulating IR. Moreover, fibroblast growth factors (FGFs) comprise a set of small polypeptide growth factors that contribute to the development of diseases. They comprise twenty-two structurally related proteins classified as endocrine, paracrine, and intracrine FGFs [99]. Li and colleagues explored the functions of FGF in high-fat diet HFD (HFD)-fed mice treated with HDAC inhibitors. They produced FGF21 KO mice that displayed glucose intolerance and obesity. This shows the significance of FGF21 in metabolic disorders [100]. FGF21 hormone has been shown to promote glucose homeostasis and lipid homeostasis. Various studies have also shown improvements in sensitizing insulin [101,102,103]. Thus, the induction of FGF21 can be a possible therapeutic strategy for treating IR and obesity, while inhibiting HDAC is one way to increase the expression of FGF21 [104].

Myocyte enhancer factor-2(MEF-2) binding domain and domain 1 mainly govern the GLUT4 gene promoter. The transcriptional activity is advanced when MEF2 attaches to the binding domain of MEF2 and GLUT4 enhancer factor (GEF) attaches to Domain 1 [105,106]. A reduction in MEF2 results in reduced expression of GLUT4 [107]. Class Ⅱ HDACs, HDAC4 and 5, inhibit the MEF2 transcriptional activity and inhibit GLUT4 expression leading to IR [108,109,110].

HDAC6 KO mice have improved insulin signaling and glucose intolerance in dexamethasone-induced diabetes and insulin-induced models. This is a clear indication of HDAC6 as an important regulator of insulin signaling [111]. The most widely used models for diabetes are the genetically modified db/db mouse and ob/ob mouse. HDAC 4/5/7 is required for the hyperglycemic state in diabetic mouse models mentioned above [112]. Mihaylova and co-workers demonstrated the role of HDACs in maintaining glucose levels in the liver of HFD-induced diabetic mice. They reported a reduction in the presence of class Ⅱa HDAC in diabetic mouse models and a reduction in blood glucose during fasting. The HFD mouse model also showed decreased fasting glycemia levels and enhanced glucose tolerance when class Ⅱa HDAC levels were reduced [112]. Wang and co-workers demonstrated an elevated expression of HDAC5 and HDAC9 in the brain of HFD-fed C57BL/6J mice. Compared to the control mice, these mice demonstrate reduced insulin sensitivity and increased levels of fasting blood glucose [113]. These studies showed links between HDAC and IR.

## 5. Linking Peripheral IR with Impaired Brain Insulin Signaling and AD

During normal conditions, insulin traverses the rigid blood–brain barrier (BBB) easily via a receptor-mediated transport mechanism. It is still not clear whether insulin is synthesized and secreted in the CNS. However, both rodent and human studies have confirmed the presence of insulin mRNA in the brain and the secretion of insulin from GABAergic interneurons and choroid plexus epithelial cells [114].

**Figure 4:**
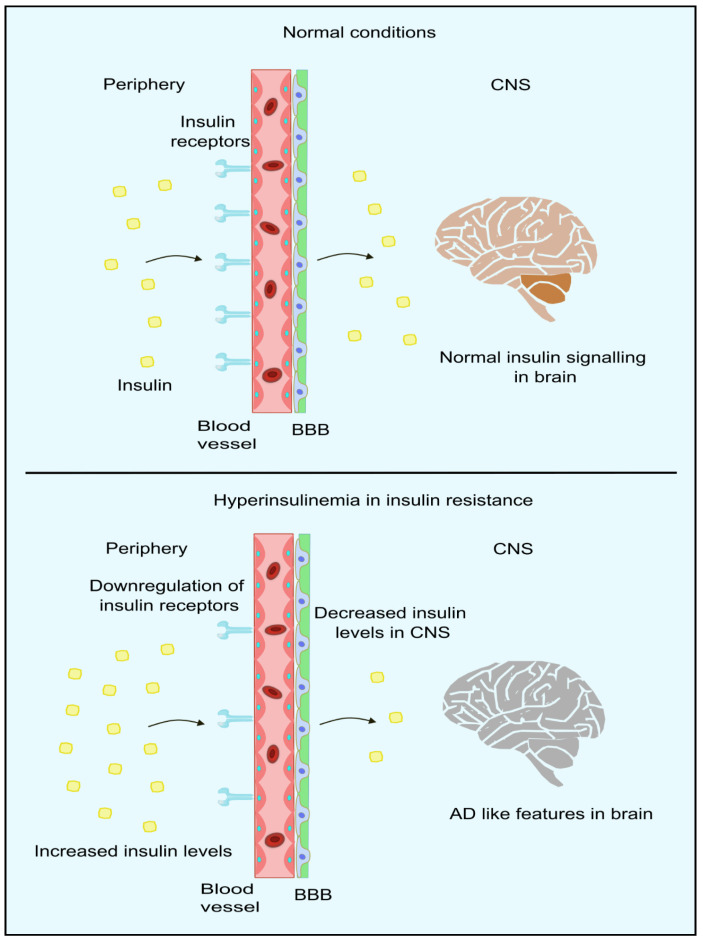
Insulin signaling in periphery and brain. In normal conditions, insulin crosses the BBB through the insulin receptors present in the BBB. However, in hyperinsulinemic conditions, insulin receptors are downregulated, resulting in lower insulin levels in CNS, thus leading to AD-like features in the brain. Created using inkscape.org (Accessed on 17 November 2023).

IR can be defined as the unresponsiveness of target tissues towards the action of insulin [114]. Peripheral IR is commonly assessed by the gold-standard assessment method of a HOMA-IR [115]. Accumulating studies have shown that IR in the periphery can result in negative impacts on the brain, which include reduced insulin uptake, rising amyloid β, pro-inflammatory cytokines, tau protein phosphorylation, oxidative stress, advanced glycation consequence, and finally, apoptosis [116,117,118,119,120,121]. It has also been proposed that the brain becomes resistant to insulin in AD with/without comorbid T2DM. This acts as a critical trigger in other pathophysiological events in this disorder [122,123,124,125]. Moreover, this is consistent with studies that report alterations in those molecules responsible for insulin signal transduction, present in the forebrain of individuals with AD. Furthermore, some studies have reported improvement in the memory of AD patients after administration of intranasal insulin [125,126,127,128,129]. The insulin receptors and insulin-dependent-GLUT is compromised in AD patients [130,131]. Moreover, peripheral IR also alters the proper functioning of CNS, such as the down-regulation of insulin transport to the brain, making the neuropathological features more evident in AD patients [132,133]. Insulin transport across BBB occurs mainly through carrier-mediated [134] and saturable processes [135]. Thus, an increase in the peripheral insulin levels, as in the case of hyperinsulinemia (mainly during IR), results in a higher amount of insulin in the cerebrospinal fluid, whereas in chronic cases, down-regulation of insulin receptors occurs in the BBB, leading to impaired insulin signaling as indicated in Figure 4 [136]. In studies where systemic insulin infusion [137,138,139,140] or intranasal insulin [141,142] is administered, a possible link between peripheral IR and brain IR was observed. However, CNS insulin sensitivity is restored through caloric restrictions for diabetic and obese patients. This improves the peripheral metabolism in diabetics and obese patients [143]. Thus, we can conclude that peripheral insulin signaling relates to brain insulin. However, it is not clear whether the resistance exists in the periphery and CNS. In the liver, insulin contributes to a significant role in inhibiting glucose synthesis as well as its release by blocking major enzymes in the gluconeogenesis and glycogenolysis pathway. Phosphoenolpyruvate carboxylase (PEPCK) is the major gene encoding the hepatic enzymes for the regulation of the pathways mentioned above. The blockage of the expression of PEPCK blocks the synthesis of glucose [144,145,146].

When insulin attaches to the α-subunit, which is present extracellularly in the insulin receptor, autophosphorylation of the tyrosine residues occurs in the cytoplasmic segment of the β-subunit, resulting in the initiation of intrinsic tyrosine kinases, catalyzing the intracellular substrate phosphorylation on tyrosine which includes the insulin substrate family, the src homology and collagen (shc) adaptor protein, Gab-1, and Cbl. Following the phosphorylation process, various src homology 2 (SH2) domain proteins, along with the other adapter molecules like Grb-2, Crk, and Nck, bind to the IRS proteins upon phosphorylation. One of the most vital SH2 proteins is the phosphatidylinositol 3-kinase (PI-3 kinase). PI3-kinase contributes to the translocation of insulin-dependent GLUT-4. PI3-kinase, once activated, generates phosphatidyl inositol phosphate-3,4,5-trisphosphate (PIP3). An increment in the levels of PIP3 results in the initiation of protein kinase cascade, with PDK being the first protein kinase to be activated. PDK phosphorylates and initiates serine/threonine kinases, including the Akt (protein kinase B), protein kinase C-PKCζ and λ. Both isoforms and Akt help mediate insulin-mediated glucose transport in muscle and adipose tissue. However, Akt appears to take part in a more critical function in glycogen production and storing glucose in muscle, adipose tissue, and liver [147].

Researchers have reported the existence of insulin receptors in certain areas of the hippocampus, cortex, hypothalamus, and the olfactory bulb, which have been shown to take part in brain insulin signaling. In the brain, insulin receptors are more widespread in the neurons and synapses compared to glial cells. Brain insulin receptors are accountable for various functions, such as maintaining synaptic plasticity, neurotransmission, homeostatic regulation, and age-related neurodegeneration [4]. In a study conducted on AD patients by Frolich et al., reduced sensitivity of insulin signaling was reported in the brain tissues, even in those patients not having T2DM [148]. A reduction in the levels of IGF 1 and 2 was observed, along with a decrease in insulin receptors, insulin-associated PI3-kinase, and the activated Akt-PkB kinase in a few studies [3]. Brain IR is a state in which the brain cells do not respond to normal levels of insulin [5]. This condition causes an increased production of amyloid β and phosphorylation of tau protein, which are the two main hallmarks of AD. Additional hallmarks such as protein misfolding, oxidative stress, and mitochondrial and cognitive dysfunction are also associated with brain IR. IR further leads to elevation in insulin levels, thus competing with amyloid β for insulin-degrading enzyme (IDE). IDE is accountable for the degrading insulin and amyloid β [149].

## 6. Advanced Drug Delivery Systems in Epigenetics

The epigenome is very complex and highly regulated in nature. The delivery and kinetics of epigenetic drugs are crucial as these are highly potent medications that can produce several adverse drug reactions when the delivery is not targeted [150]. An ideal delivery system for epigenetics drugs should have the ability to retain the stability of the drug as well as provide a targeted delivery in a controlled manner. The pharmacokinetic studies of epigenetic drugs can be utilized for the design of an effective delivery system. For instance, the presence of enzymes can result in alteration in the ADME processes [151]. As these drugs generally do not bind to plasma proteins, a higher risk of renal clearance is present, which can result in the need for a delivery system that can continuously deliver the drug only to the desired location of action at a predetermined rate. Thus, there is a significant need for a drug delivery system for the delivery of epigenetic drugs.

Various types of delivery systems can be used for targeted delivery, such as nanoparticle-based technologies, vesicular systems, network systems, biological vectors, etc. Strategies such as stimuli-based delivery systems (heat, light, enzymes, mechanical force, magnetic field, etc.), synergistic drug combinations, and prodrug approaches can also be utilized to enhance drug delivery [152]. The selection of a vehicle or system is very important for the success of delivery. It will depend greatly upon the physiochemical properties of the drug and the pathological conditions of the patient [153]. The various types of drug delivery systems used for the delivery of epigenetic drugs mainly include nanoparticle-based drug delivery systems, vesicular systems, engineered nano-carriers, network systems, hydrogels, as well as biological vectors.

### 6.1. Nanoparticle-Based Systems

Use of nanoparticles (NPs) provides a versatile delivery system for brain delivery of epigenetic drugs due to their characteristic properties, such as smaller particle size, surface morphology, zeta potential, chemical composition, solubility, and functionalization [154]. Various types of polymers, polysaccharides, and proteins can be used for the synthesis of NPs, such as poly lactic-co-glycolic acid (PLGA), dextran, albumin, polyethylene glycol (PEG), gelatin, chitosan, and inorganic NPs such as silica, gold, zinc oxide, and silver particles. These particles are used for the delivery of both chemical moieties and peptides in chronic neurodegenerative diseases such as AD, autoimmune diseases such as multiple sclerosis and rheumatoid arthritis, as well as Parkinson’s disease [155,156,157]. NPs can be chemically modified and functionalized to traverse the BBB and effectively deliver the drug to the CNS [158]. PLGA NPs are regarded as a promising system for AD, with their property to produce a site-specific delivery [159]. Studies have proved that curcumin-loaded PLGA-based NPs conjugated with peptides can decrease the disease progression in AD [160]. It was also found that PLGA NPs when loaded with vitamin D binding proteins, suppress the symptoms of AD [161]. For uniform brain distribution, albumin-loaded NPs are preferred. Bovine serum albumin NPs combined with cyclodextrins are used for the intranasal delivery of tacrine in AD [162]. Mesoporous silica-based systems are used to produce a stimulus-based controlled delivery in AD [163]. Apart from all this, NPs can be utilized in theranostic applications [164,165,166]. For instance, chitosan–hyaluronic acid NPs are used as targeting agents in AD with additional theranostic properties [167]. NP-based systems are highly biocompatible and biodegradable, making them a versatile system for targeted drug delivery in neurodegenerative diseases.

### 6.2. Vesicular Systems

Vesicular systems widely utilized in the case of drug delivery include liposomes, niosomes, cubosomes, pharmacosomes, aquasomes, phytosomes, transferosomes, and electrosomes. These are self-assembling systems that are mainly composed of lipid bilayers. They can form a core and an outer layer, which enables them to encapsulate and deliver both hydrophilic and hydrophobic drugs. The release of drugs from these systems can be controlled by engineering the system and by using varying concentrations of lipids for the synthesis of the outer lipid layer. Surface functionalization combined with structural modifications of vesicular systems is extensively used for localized delivery and drug-targeting applications [168].

Liposomal systems are the frequently used delivery system for neurological disorders due to their potential to traverse through the BBB. In the past few decades, intranasal delivery of liposomes was explored for the treatment of AD [169]. It was reported that surface-functionalized liposomes can reduce the β-amyloid burden and reduce the progression of AD [170]. Liposomes can also be synthesized with the same lipid composition as that of exosomes, thereby, reducing the heterogenicity of naturally occurring exosomes, making it an innovative carrier that has high productivity [171]. Bifunctional niosomes have also been developed for AD that can be delivered intranasally to reduce drug wastage by first-pass metabolism [172]. Cubosome is a novel nano-carrier used for brain delivery that can cross the BBB and control the delivery rate. Nanostructured in situ gels of cubosomes are used for the delivery of AD medications. These systems have been utilized in other neurological diseases, such as epilepsy and dementia, for effective treatment [173,174]. Oral nanomedicines using cubosomes have been developed with piperine and tween for the treatment of AD. These systems use bioactive excipients for the oral delivery of AD medications [173].

### 6.3. Network Systems and Dendrimers

Interpenetrating polymeric network systems is one of the widely used strategies adopted for the delivery of epigenetic drugs [175]. Over the past few decades, scientists focused on the swelling properties, stability, and biocompatibility of these polymers to produce a biodegradable delivery device [175]. The advanced form of this network system is dendrimers with well-defined structures and shapes. The multivalency of these nanostructures is extensively utilized for the delivery of drugs, proteins, peptides, and genes [150]. They are made up of functionalized monomers that form the nano-architecture for the conjugation of molecules and their delivery [176]. Studies have proved that poly(amidoamine) (PAMAM)-based dendrimers not only deliver drugs in a controlled manner but also provide neuroprotective action [177]. It has also been reported that cationic phosphorus dendrimers can act synergistically with AD medications [178].

### 6.4. Hydrogel-Based Systems

Hydrogel-based nano-formulations are gaining interest due to their ability to control the degradability of the system and produce a desired drug-release pattern. One of the most advanced hydrogel systems is the use of nanogels, which are developed to release therapeutic drugs in response to a particular stimulus. One of the major advantages of a nanogel is its high biodegradability, biocompatibility, and lower toxicity in the in vivo system [179]. When the drug to be delivered is highly lipophilic, another nano-formulation known as the solid-lipid nano-carrier system is used [180]. The potential of these systems to traverse through the BBB helps the drug to reach the brain cells easily. These nanostructured lipid carriers can also be further engineered to receive an optimum delivery pattern near the targeted cells [181]. A hydrogel micro post-based system has been developed for the detection of microRNA (miRNA) that are associated with AD. These systems use PCR techniques for early diagnosis of the disease [182] and are highly recommended in AD drug delivery owing to their properties to induce neurite outgrowth and neuroprotective actions [183].

### 6.5. Biological Vectors

Nanocells have been recently used for the delivery of biological products. Chemical moieties can also be loaded into these cells for targeted delivery. These bacterial cells are devoid of genetic material and cannot replicate on their own, thus maintaining the dose that is initially administered. The inability of these cells to produce mutations increases the acceptance of these bacterial cells for human use [150]. They can also deliver genetic materials as they provide a stable environment for RNAs and DNAs. A major risk produced by nano-cells is the higher chances of immunogenicity caused due to the presence of lipopolysaccharide [184]. Further research should be carried out to optimize the use of biological vectors for epigenetic drug delivery. Organoids have gained much interest among scientists for the development of a qualified model for AD [185]. Cerebral organoids are mainly used in culturing and modeling of phenotypes like AD. Brain organoids are more advantageous as compared to other AD models due to their ability to form a next-generation humanized disease model [186]. Organoids have not only been developed for AD but have also been cultured for other neurological diseases such as amyotrophic lateral sclerosis, Parkinson’s disease, and Huntington’s disease. Various other types of therapies include the use of gene therapy [187], mRNA [188], siRNA [189], miRNA [190], and antisense oligonucleotides [191].

## 7. Conclusions

In this review, we attempted to explain how epigenetic modifications can impact the insulin levels in the periphery to cause peripheral insulin resistance. Long-term peripheral insulin resistance leads to type 2 diabetes mellitus and further complications. Type 2 diabetes mellitus is the trigger for most metabolic disorders like hypertension, metabolic dysfunction associated with steatotic liver disease, metabolic dysfunction associated with steatohepatitis, hyperlipidemia, etc. Studies have also shown that insulin resistance influences the brain insulin signaling and causes insulin resistance in the brain. Insulin in the brain competes with amyloid-β for insulin degrading enzyme, thus causing Alzheimer’s disease, also known as type 3 diabetes mellitus. We also discussed the various advanced drug delivery systems that can be used to counter the epigenetic changes leading to AD. The various drug delivery systems discussed in this paper (nanoparticles, vesicular systems, hydrogel-based systems, and biological vectors) possess unique characteristics that make them suitable and a potential option for targeting drugs to the brain. With the addition of biotechnology into these methods, the above-mentioned drug delivery systems pave the way for newer and technologically advanced systems for not only treating Alzheimer’s disease but also other neurological diseases such as Huntington’s disease, Parkinson’s disease, and amyotrophic lateral sclerosis.

## Abbreviations

Aβ	Amyloid β
AD	Alzheimer’s Disease
ATF-2	Activator Transcripton Factor-2
BBB	Blood Brain Barrier
CRE	cAMP Responsive Element
CREB	cAMP Responsive Binding Protein-1
CRB	CREB Binding Protein
Dnmts	DNA methyl transferases
FADS	Fatty Acid Desaturase
FGF	Fibroblast Growth Factor
GCK	Glucokinase
GLP-1	Glucagon Like Peptide-1
HAT	Histone Acetyl Transferases
HDAC	Histone Deacetylases
HFD	High Fat Diet
IDE	Insulin Degrading Enzyme
IGFBP	Insulin Like Growth Factor Binding Protein
IR	Insulin Resistance
KO	Knock Out
MEF-2	Monocyte Enhancer Factor-2
ncRNA	non-coding RNA
NP	Nano Particles
NT	Neurofibrillary Tangles
SiRNA	Short Interfering RNA
T2DM	Type 2 Diabetes Mellitus
